# The impact on patients of objections by institutions to assisted dying: a qualitative study of family caregivers’ perceptions

**DOI:** 10.1186/s12910-023-00902-3

**Published:** 2023-03-13

**Authors:** Ben P. White, Ruthie Jeanneret, Eliana Close, Lindy Willmott

**Affiliations:** Faculty of Business and Law, Australian Centre for Health Law Research, Queensland University of Technology, PO Box 2434, Brisbane, QLD 4000 Australia

**Keywords:** Assisted dying, Medical assistance in dying, Euthanasia, Assisted suicide, Institutional objection, Patient experience

## Abstract

**Background:**

Voluntary assisted dying became lawful in Victoria, the first Australian state to permit this practice, in 2019 via the *Voluntary Assisted Dying Act 2017* (Vic). While conscientious objection by individual health professionals is protected by the Victorian legislation, objections by institutions are governed by policy. No research has been conducted in Victoria, and very little research conducted internationally, on how institutional objection is experienced by patients seeking assisted dying.

**Methods:**

28 semi-structured interviews were conducted with 32 family caregivers and one patient about the experience of 28 patients who sought assisted dying. Participants were interviewed during August-November 2021. Data from the 17 interviews (all with family caregivers) which reported institutional objection were analysed thematically.

**Results:**

Participants reported institutional objection affecting eligibility assessments, medication access, and taking the medication or having it administered. Institutional objection occurred across health settings and was sometimes communicated obliquely. These objections resulted in delays, transfers, and choices between progressing an assisted dying application and receiving palliative or other care. Participants also reported objections causing adverse emotional experiences and distrust of objecting institutions. Six mediating influences on institutional objections were identified: staff views within objecting institutions; support of external medical practitioners and pharmacists providing assisted dying services; nature of a patient’s illness; progression or state of a patient’s illness; patient’s geographical location; and the capability and assertiveness of a patient and/or caregiver.

**Conclusions:**

Institutional objection to assisted dying is much-debated yet empirically understudied. This research found that in Victoria, objections were regularly reported by participants and adversely affected access to assisted dying and the wider end-of-life experience for patients and caregivers. This barrier arises in an assisted dying system that is already procedurally challenging, particularly given the limited window patients have to apply. Better regulation may be needed as Victoria’s existing policy approach appears to preference institutional positions over patient’s choice given existing power dynamics.

**Supplementary Information:**

The online version contains supplementary material available at 10.1186/s12910-023-00902-3.

## Background

There is an international trend to legalise assisted dying (“AD”), also known as medical assistance in dying, physician-assisted suicide and euthanasia [1]. Despite being lawful in many jurisdictions globally, AD remains controversial. Generally, health professionals can refuse to participate in AD through conscientious objection. While the appropriate scope of such objection remains contested, it is a well-recognised concept with protections in law and policy [2].

Healthcare institutions may also object to AD, yet how rights and obligations of objecting institutions are, or should be, conceptualised is less clear. While some analogies with individual conscientious objection are possible [3], there are important differences, including for example, the prospect of a wider access barrier for patients when an entire institution, as opposed to an individual health professional, objects to AD [4]. There may also be more diverse grounds for healthcare institutions to object.

Such objections are typically claimed by faith-based institutions, predominantly Catholic ones, which commonly provide a large proportion of end-of-life care [3, 5, 6]. But some argue institutions are incapable of holding a moral or ethical position based on conscience [3, 7]. An institution is a corporate organisation that, unlike an individual, cannot experience guilt or suffer moral injury from acting against its conscience. Others contend institutions may have a distinct mission and moral identity [6]. For example, Catholic institutions are centred on an “ethic of care” and Catholic values, an ethos which some argue is analogous to an individual’s conscience [3, 6].

In a somewhat different vein, Shadd and Shadd skirt the debate about the existence of institutional conscience and instead contend institutions’ right to object is a matter of self-governance, provided they have a “legitimate reason” for the objection, including moral or religious justifications [8]. Others respond that institutional objection must be curtailed and balanced against protecting patient interests, given the considerable harms such objections can cause [3, 9, 10]. This is important because patients seeking AD are often vulnerable by virtue of disease, illness, and/or frailty, and existing power asymmetry with institutions is more pronounced [9].

Despite bioethical engagement with institutional objection, there is limited empirical research on its impact on AD [10–13]. Studies report on objections by institutions to providing information about AD, eligibility assessments, and provision of AD medication onsite [12, 13]. In some cases, objections have resulted in forced transfers out of a facility for an AD assessment or provision, causing additional pain, suffering, and stress for patients and caregivers [10, 12–14]. In other cases, institutional objections have precluded access to AD because a transfer is unavailable or physically unbearable [4, 10, 13]. The literature also suggests broader impacts of institutional objection, including it being a risk factor for complicated grief [15], and “knock-on” effects of an institution’s policy affecting the willingness of local healthcare professionals to participate in AD [12]. Existing findings about institutional objection have usually been included as part of wider reports about patients’, caregivers’, or health professionals’ perspectives on AD more generally, and therefore the discussions of institutional objections are brief. As an increasing number of jurisdictions legalise AD, more research is needed to better understand how institutional objection can arise, the factors affecting patient experiences, and the impact of the particular regulatory context.

This article helps address this knowledge gap. It reports on institutional objection to AD in Victoria, Australia and draws on the country’s first study of patient AD experiences, as reported by family caregivers. Victoria is examined as it was the first Australian state to legalise AD. Its *Voluntary Assisted Dying Act 2017* (Vic) has been operational for over three years. The Act’s default method of AD is self-administration, where patients take the medication themselves (physician-assisted dying), but practitioner administration, where the medication is administered by a doctor (euthanasia) is permitted when patients are not physically capable of taking or digesting the medication [16]. Eligibility criteria include that a patient is terminally ill with doctors required to confirm that a patient is expected to die within 6 months, or 12 months for neurodegenerative conditions.

On the issue of institutional objection, the Victorian legislation is silent, an approach followed in the other states of Western Australia and Tasmania. By contrast, the legislation in South Australia, Queensland and New South Wales (the last three Australia states to legalise AD) specifically regulate institutional objection, with varying balances struck between ensuring patient access and respecting institutions’ positions.

The legislative silence on institutional objection in Victoria led to regulation via policy. The Department of Health issued policy recommendations [17] and each institution manages its own institutional position and local policy development. The Department’s policy guidance is permissive in that it suggests models of participation and possible steps, such as referrals to a statewide AD navigation service to facilitate access, but it does not require institutions take particular steps. Reflecting this, an analysis of publicly-available AD policies produced by objecting institutions demonstrated they contained little practical guidance that would assist patients to navigate those objections to AD [18].

This article reports on how institutional objection has manifested in practice, including the nature of objections expressed, practices institutions prohibited, views of employed staff, impact on patients, and mediating factors affecting patient experiences.

## Methods

### Research design

This study is part of a broader research project, involving interviews with patients, family caregivers, health professionals and regulators in Australia (as well as two case study countries, Canada and Belgium) [19]. The project seeks to understand participants’ perspectives and experiences of decision-making about AD and how regulation is working in practice, to inform an optimal holistic model of regulation [20]. This article focuses on patient experiences of institutional objection while seeking AD, in the Australian state of Victoria, as reported by family caregivers.

We adopt a critical realist approach to this research, [21] and used Braun and Clarke’s reflexive thematic analysis [22]. As noted below, our reflexive practice [22] included BPW and RJ conducting these interviews together, and debriefing after each interview as well as periodically discussing with the authorial team the initial analysis and interpretations of the data. A research journal was maintained and referred to throughout the data collection and analysis processes. All authors reviewed the final data collected and interpretations were shared and iteratively discussed to achieve a richer understanding of the data [23]. The method is reported according to the Consolidated Criteria for Reporting Qualitative Research [24].

### Sampling and recruitment

For the wider study investigating patients’ experiences of seeking AD in Victoria, Australia, participants eligible for inclusion were patients seeking AD in that state, and family caregivers who had or were supporting patients through this process. “Seeking AD” meant that the assessment process had started, but it did not have to be completed, nor did the person have to be approved for AD to be eligible to participate in this study. Participants had to be over 18 years of age.

As discussed further below, we were only able to recruit one patient in the broader study, and they did not experience institutional objection, hence this article is based solely on reports of patient experiences by family caregivers as proxy. While accounts directly from patients would have been preferable, to be eligible for AD in Victoria, patients must be terminally ill (within 6 or 12 months of death depending on their condition) and suffering intolerably, making this a challenging cohort to recruit. Many participants are too ill to participate in research once a terminal prognosis is established [25]. Challenges with recruitment of terminally-ill patients are well-recognised in end-of-life research, and after-death interviews with family caregivers are the next best way to explore patient experience [25, 26].

Recruitment occurred through social media (Twitter) and key patient interest groups Go Gentle Australia and Dying with Dignity Victoria (sharing study details via social media, newsletters, and direct emails). Initially relying on convenience sampling, we later used purposive sampling seeking a breadth of domains including patient age, sex, illness, location (metropolitan/regional), timing of seeking access, and patient experience of AD (self-administration, practitioner administration, sought AD but did not use or not approved). These later recruitment communications specifically stated the particular patient characteristics we were yet to collect data on, and this included direct emails from some of the patient interest groups noted above to potentially matching participants.

### Data collection

An interview guide (Additional File 1) was developed based on our analysis of the Victorian legislation [16], previous interviews with doctors [27–29], and discussion within the research team. Key areas explored were: process of seeking AD including seeking information, eligibility assessments, and accessing and taking medication or having it administered; navigating the system; and overall perceptions of the system’s operation. For cases when an institution objected to AD, discussion of this was often initiated by the participant in the course of explaining the patient’s experience of the AD process. But a more general question was also asked if this issue was not specifically raised: “Did the facility facilitate access to AD or was it a barrier to access?”. When an institutional objection was reported, follow up questions explored issues such as: the stage in the process where barriers arose (e.g. when AD was first raised, during eligibility assessments or at the medication stage); what the impact of the objection was; and the role of institution staff in implementing and communicating the objection.

Interviews traced the patient journey of seeking AD. Family caregivers were asked to report their perceptions of the patient’s AD experience, but they also shared their personal views and experience. For example, when participants described the impact of institutional objection on them and family members other than the patient, these experiences were explored. Most of the caregivers interviewed had accompanied their family member patient throughout their AD journey, for example, caring for them at home and being present during medical appointments or clinical discussions in hospitals or other facilities, and so were able to draw on this shared experience.

Participants provided free and informed consent. For all family caregivers, the patient whose experience they were sharing had died, and so patient consent was not sought. Some interviews involved two participants at their request, for example, two children of a deceased patient. All interviews were conducted by two authors BPW and RJ together, with one a designated lead. Interviews occurred between 17 August and 26 November 2021 via Zoom video conferencing except for two by phone and one in-person. Recruitment ceased once the research team considered there was sufficient “information power” to meet the study aims [30]. Interviews were digitally audio-recorded and transcribed verbatim. Participants had an opportunity to amend or add to their transcript (member checking) [31] and some provided additional supplementary information (e.g. chronology or narrative of patient experience).

### Analysis

Analysis occurred in two main stages. The first involved thematic analysis of transcripts and participants’ supplementary information line by line with codes developed both deductively (from literature and iterative discussion of emerging themes) and inductively [22]. Seventeen interviews were double coded by BPW and RJ (codes discussed and refined periodically), with BPW coding the remainder. Iterative analysis occurred while collecting data with BPW and RJ debriefing after each interview, and regularly throughout data collection and analysis. This first stage of analysis included identifying those interviews that reported institutional objection, namely when a participant perceived an institution objected to some or all aspects of AD, including when this objection may not have been expressly stated. This included where participants reported perceiving access to AD would be affected because of an institution’s stated religious affiliation, or because of interactions with an institution's staff, even if it was not expressly stated that AD would not be permitted.

The second stage involved a focused analysis of this subset of interviews reporting institutional objection. Using reflexive thematic analysis, BPW recoded this data inductively line by line to develop further sub-themes about how patients experienced institutional objection [22]. These preliminary findings were iteratively discussed by all authors, who also studied all institutional objection data, to enhance the richness of analysis. This second stage included reviewing transcripts as a whole to understand institutional objection in context (e.g. impact of geographic location, nature of illness, timing of AD experience). Both stages of analysis were aided by NVivo (release 1.6.1 QSR International) which was used to store, code, and search transcripts.

## Results

Twenty-eight interviews were conducted with 32 family caregivers and one patient (Table 1) in relation to the experiences of 28 patients (Table 2). The sole patient interview involved a participant who spoke about their own experience of seeking AD. In the remaining family caregiver interviews, participants reported on the experience of their family member as a patient seeking AD, all of whom were deceased at the time of interview. The median length of interviews was 90 min, with a range of 56 min to 130 min.

**﻿Table 1:** Characteristics of interview participants (total sample and institutional objection study sample)

Characteristics	Total sample (n = 33): number (n %)	Institutional objection study sample (n = 20): number (n %)
Age (years)	Mean: 56.6	Mean: 56.9
20–29	1 (3%)	0 (0%)
30–39	4 (12%)	2 (10%)
40–49	7 (21%)	5 (25%)
50–59	3 (9%)	3 (15%)
60–69	13 (39%)	7 (35%)
70–79	4 (12%)	3 (15%)
80–89	1 (3%)	0 (0%)
Sex		
Female	26 (79%)	18 (90%)
Male	7 (21%)	2 (10%)
Relationship to patient*		
Child (including stepchild, child in-law)	17 (50%)	12 (60%)
Spouse (including de facto partner)	10 (29%)	6 (30%)
Parent	3 (9%)	2 (10%)
Sibling	2 (6%)	0 (0%)
Close friend	1 (3%)	0 (0%)
Self	1 (3%)	0 (0%)

*One participant in the overall sample spoke about two patients so is included in two categories. Percentages in that section of the table are calculated using number of relationships (34)

**Table 2:** Characteristics of patients whose voluntary assisted dying experience was the subject of interviews (total sample and institutional objection study sample)

Characteristic	Total sample (n = 28): number (%)	Institutional objection study sample (n = 17): number (%)
Age (years)	Mean 70.8	Mean 73.2
20–29	1 (4%)	0 (0%)
30–39	1 (4%)	1 (6%)
40–49	0 (0%)	0 (0%)
50–59	3 (11%)	1 (6%)
60–69	7 (25%)	4 (24%)
70–79	8 (29%)	5 (29%)
80–89	6 (21%)	5 (29%)
90–99	2 (7%)	1 (6%)
Sex		
Female	13 (46%)	7 (41%)
Male	15 (54%)	10 (59%)
Location		
Metropolitan	16 (57%)	13 (76%)
Regional	12* (43%)	4 (24%)
Highest level of education		
Some high school	7 (25%)	6 (35%)
High school	9 (32%)	4 (24%)
University–diploma	1 (4%)	0 (0%)
University–undergraduate	7 (25%)	4 (24%)
University–postgraduate (including graduate diploma)	4 (14%)	3 (18%)
Primary disease, illness, or medical condition		
Cancer	18 (64%)	10 (59%)
Neurological	9 (32%)	6 (35%)
Other	1 (4%)	1 (6%)

Eligibility for voluntary assisted dying and death		
Assessed as eligible	24 (86%)	16 (94%)
Patient died via self-administered medication	19 (68%)	12 (71%)
Patient died via practitioner administered medication	3 (11%)	3 (18%)
Patient died but did not take medication (natural death)	1 (4%)	1 (6%)
Patient waiting to take medication	1 (4%)	0 (0%)
Patient died prior to eligibility assessment completed	3 (11%)	1 (6%)
Patient assessed as ineligible and died	1 (4%)	0 (0%)

Timing of voluntary assisted death (or engagement with process)		
July–December 2019	4 (14%)	2 (12%)
January–June 2020	6 (21%)	4 (24%)
July–December 2020	3 (11%)	1 (6%)
January–June 2021	10 (36%)	6 (35%)
July–November 2021	5 (18%)	4 (24%)

*One patient in the overall sample who was classified as regional moved to a metropolitan area during the voluntary assisted dying process.

Seventeen of the 28 interviews (Table 1) discussed an institutional objection (distinct from conscientious objection by an individual) impacting on patient access to, or experiences of, AD. These 17 interviews were all with family caregivers (n = 20), and related to the experience of 17 patients (Table 2). The median length of these interviews was 95 min, with a range of 56 min to 130 min. The remaining eleven interviews that did not consider institutional objection are not included in this further analysis.

A broad range of themes were identified: the basis and expression of the objection; nature of the AD-related activity objected to; impact of institutional objection; spectrum of staff views within objecting institutions; and factors mediating the impact of institutional objection.

### Basis and expression of institutional objection

Participants principally cited Catholic institutions as manifesting objections to AD (Box 1). Some also saw palliative care philosophy as founding objections. Sometimes these grounds overlapped. Objections occurred across public and private healthcare settings and by hospitals, palliative care units, residential aged care facilities and community care organisations.

How and when an institutional objection was expressed varied. Some institutions made “explicit statements from the start” to patients and/or caregivers. But one participant reported being surprised because a clear direction that the AD medication could not be taken onsite was communicated to them and the patient very late in the process. Some stated they already knew an institution’s objection through media statements or published policy positions.

Other times, an institution’s objection was gleaned only through context and interactions and not explicitly stated. One participant spoke of just getting “a sense” a transfer of the patient would be needed. Sometimes participants inferred AD was off-limits because of religious affiliation: “it’s a Catholic place”.

Box 1: Participant quotes—Basis and expression of institutional objection Many of the palliative care organisations are run by Catholic institutions who are not in favour of voluntary assisted dying. So that was always going to be a bit of an issue for us in talking about it with [patient name]… (Family caregiver of patient with cancer)

It was in the media. Catholic-based health facilities put out a joint statement, and their joint statement was that they were conscientious objectors to the voluntary assisted dying. (Family caregiver of patient with cancer)

I knew for a start that she couldn’t die there… because I’d looked it up on their website. So we didn’t even pursue it. So I always knew that it wasn’t going to happen. (Family caregiver of patient with neurological condition)

### Practices institutions objected to

Many objecting institutions prohibited most or all of the AD process. Participants’ reports centred on three key aspects (Box 2). The first was not permitting AD eligibility assessments within the institution. Particular hospitals were described as barring entry to outside doctors attending to assess a patient’s eligibility. The second was precluding receipt of the medication. Some institutions denied access to the Statewide Pharmacy Service, which delivers the medication to eligible patients. The third was not allowing AD medication to be taken or administered onsite. For in-patients or those in residential care, this meant having to be discharged or transferred to access AD. In one case, the institution would allow self-administration, but not practitioner administration, onsite. In another case, for a patient at home, a community care nurse was prohibited by her employer from being present when AD occurred.

In addition to these three key aspects of the AD process, participants also gave examples of staff not being allowed to discuss AD with them or patients, refusing admission to a residential facility for a patient intending to seek AD, and concerns about death certification.

Box 2: Participant quotes—Practices institutions objected to [T]he oncologist said he would come to the hospital to do the second appointment, and when he heard I was at [Catholic hospital], he said, “Oh, sorry, I can’t come there, you’ll have to wait for [patient name] to come out of hospital.” (Family caregiver of patient with cancer)

That was at the time when it was likely that Mum was going to be transferred to [Catholic hospital] because her pain was so severe… [Catholic hospital] actually told us that if she came … that they would not allow the state pharmacist representatives to come into the hospital at all. (Family caregiver of patient with neurological condition)

They said, “… so do you think you’d like to go [home–town name] or is there somewhere else?” Dad said, “No, I will just do it here.” At that point they said “Well, actually we can’t do it on hospital grounds.” So Dad [said], “Well, okay, push me out to the carpark and I’ll do it there.” (Family caregiver of patient with cancer)

I was saying that “I’m actually really hoping that she goes down this avenue,” and … she said, “I have to stop you there … I’m so sorry, but because of where I work I’m actually not allowed to actually have a conversation with you about assisted dying at all.” (Family caregiver of patient with neurological condition)

### ﻿Impact of institutional objection

Participants described three key ways that institutional objection hampered patient access to or experience of AD: patient delay in accessing AD; reduced choice for patients about AD; and emotional and relationship costs for patients and family caregivers (Box 3).

Delay for patients was the principal impact, either due to prohibiting access to doctors and pharmacists, or making patients wait until they left the institution to receive or take the medication.

Patient choice was also affected. Participants described patients needing to choose between progressing the AD process or being admitted to an objecting hospital to manage pain and symptoms. In terms of missing out, one of the three patients in this study who died while seeking AD experienced an institutional objection. Their participating family caregiver considered the objection contributed to this and delays occurred because eligibility could not be assessed in that faith-based hospital. However, access to AD for this patient was challenging because of rapid illness progression and other factors.

Institutional objection also affected patient choice about place or time of taking AD medication. Participants described transfers or patients getting “shipped to a completely different hospital” or facility, including away from staff who had been caring for the patient. This often meant waiting until a bed was available in the transferring facility, resulting in delays accessing AD. Sometimes a transfer was needed back to a patient’s or family’s home to take the medication, which was not the patient’s preferred place to die.

Participants described emotional and relationship costs to patients (and families) of access being hampered by institutional objection. Both patients and families experienced anger and frustration at being in a holding pattern of not being able to seek AD. Some patients were fearful of missing out on their choice. One participant described her mother being “absolutely terrified that they would find out and … try and stop her.” Others described the stress for patients and family of uncertainty and the extra steps associated with arranging transfers to take the AD medication, impacting on what should have otherwise been a special day. Some expressed “great sadness” about patients not being able to die in a residential facility which was their home, or at the time they wanted to, or say goodbye to favourite staff. One participant described her feeling of stigma and that the family were doing something “illegal”, because of the institutional position.

There were also costs to the relationship between patient-caregiver and the treating institution with some participants reporting distrust from both caregivers and patients, with “question marks over motivations”. Against this “background of AD”, there was a loss of confidence or trust in medical advice with one participant asking, “What’s their agenda?”. Another described removing all traces of AD to ensure the death could be verified by the palliative care team.

Box 3: Participant quotes—Impact of institutional objection If we had been able to begin the access in the hospital, maybe she would have then come home and been able to complete that at home. You know, actually take part in AD at home within a day of coming home, rather than having to prolong it. [long pause] So I think institutional policies have a part to play. So allowing free access to AD doctors to access patients, if that’s what the patient wants, while they’re in hospital. Because some people spend an awful long time in hospital… (Family caregiver of patient with neurological condition)

[T]hat was a significant challenge and just created a whole lot of stress on what was her last day. You know, it was this frantic rush and … then having to wheel her out and she couldn’t say goodbye to people. … you get to the top of the mountain and then you’ve got that last big, huge boulder to climb over. It will always be a great sadness for me that the last few precious hours on Mum’s last day were mostly filled with stress and distress, having to scurry around moving her out of her so-called “home”. (Family caregiver of patient with neurological condition)

I spoke to the doctor who had helped us with the AD application … saying that [Catholic hospital] had really wanted the cognitive assessment. He said to me, “Do not let them do a cognitive assessment… you don’t know what it’s going to be used for.” I actually went back to [relevant staff member] at [Catholic hospital], who was absolutely lovely, and I said, “Look, this is what the doctor has said … I’m sure that’s not your motivation,” but s/he actually said, “I can’t guarantee that it may be somehow linked in with the AD process. So why don’t we not do the cognitive assessment.” … So s/he … didn’t say like that was the agenda. S/he just said, “Look, this whole thing is so grey and so new … I would not want to be doing anything that could potentially jeopardise your Mum’s choice in the AD thing.” So s/he was actually the one that said, “We’re just not going to do it.” [Mum] was still able to make her own decisions, but it definitely impacted what services we got from [Catholic hospital]. Because it was always in the background of AD. What’s their agenda? [C]ould it jeopardise it? And even [Catholic hospital] couldn’t say that it wouldn’t be used for something like that. (Family caregiver; no patient illness information provided to protect participant)

### Position of staff within objecting institutions

Participants described staff within objecting institutions as having a spectrum of positions on AD (Box 4).

Some staff personally objected to AD, and this exacerbated the barrier for patients. Some participants also perceived, in addition to implementing institutional objection, some staff may have been “pushing … against the AD process” towards non-AD treatment options, or advocating for admission to care locations where AD was not possible to halt the AD process. Other staff were described as simply accepting their institution’s position that AD was “out of bounds” and passively implementing that view, including by declining to discuss it.

However, participants also identified staff who they perceived disagreed with their institution. Participants described this sometimes manifesting itself as an acknowledgement of the patient’s AD choice or private moral support, communicated to both patients and caregivers. Rarely, staff went further and provided support (generally surreptitiously) for the patient to navigate towards AD.

Box 4: Participant quotes—Position of staff within objecting institutions [The staff member] said, “I can’t, because of my role, there’s nothing I can do to help you, I can’t do anything.” But she said, “I’m here for you.” (Family caregiver of patient with neurological condition)

There are people that work for [Catholic palliative care organisation] that don’t support it, but there are plenty of people that do. So I think they said, “We’ll make a note of it. But even the people that don’t support it will still care for you.” (Family caregiver of patient with cancer)

Quietly in the room. Not with other people listening. So it would be a one-on-one conversation and they said to me, “You can certainly take care of [patient name] at home. There’s no issue about that. It would be better for her if [patient] is at home.” So they were very sympathetic. They also helped direct us to a private company. Which [long pause] … that private company knew of that particular [staff member], because they said, “[Staff member] is very sympathetic and very helpful to a lot of people.” So [they] had a reputation, you would say, for looking after the patient’s needs versus the institution. Which if [they] got found out, [they] would lose [their] job. [long pause] … I'd be happy for that story to be included if I could be sure that [they were] protected. (Family caregiver; no patient illness information provided to protect participant and another person)

### Mediating influences on the impact of institutional objection

Six factors mediated the impact of institutional objection (Box 5). The first was staff views about AD which, given its broader significance, is a standalone theme above. The impact of institutional objection was felt more acutely by patients when staff shared that position, but mitigated when staff disagreed with it.

A second mediating factor was the support of medical practitioners coordinating the AD process (external to the institution) and the Statewide Pharmacy Service. Participants described these individuals making particular efforts to facilitate access despite institutional objections. Examples were pharmacists fast-tracking appointments to deliver medication before a patient’s admission to an objecting hospital or busy medical specialists doing home visits.

A third factor was the nature of the patient’s illness. Institutional objection was more problematic if a key treating hospital for a patient’s illness opposed AD. This was mentioned particularly for neurological conditions.

A fourth factor was the progression or state of a patient’s illness. If their illness was so advanced or their need for pain and symptom management required either being admitted into an objecting institution, or staying at an objecting institution where they were already receiving care, this impeded access more than for those who were able to leave the institution or remain at home. A transfer for such patients to another facility was sometimes an option but often this was unsatisfactory, for example because their illness was best treated at their existing hospital or because a bed was not available elsewhere. Further, for patients whose illness was more progressed, any delay was experienced more acutely because of shorter time they had to navigate through the AD process.

Fifth, a patient’s geographical location affected the impact of institutional objection. If there was only one health service in an area and it restricted AD, access was more complex. One participant described that a patient’s inability to self-administer would require them to transfer away from the region to receive practitioner-administration as the local institution would not permit this.

A final mediating factor was a capable and assertive patient and/or family caregiver. Objecting institutions could be identified and navigated away from (particularly in the residential aged care setting): “the place was chosen because they would allow it [AD]”. Assertive patients and caregivers also seemed more ready to challenge institutions or step past objections and seek AD privately. Some caregivers also described being able to bypass institutional objection through providing care at home, but this was not always possible. However, even participants who were highly competent and educated caregivers with health professional backgrounds found navigating institutional objection challenging.

Box 5: Participant quotes—Mediating influences on the impact of institutional objection [T]here was … a huge conflict of interest. That the AD process is not supported by [Catholic hospital] and yet [Catholic hospital] is the hospital that you go to with motor neurone disease. (Family caregiver of patient with neurological condition)

So then we had to call the State Pharmacist and say, “You need to come really, really quickly because Mum’s about to be transferred to [Catholic hospital] and you're not allowed in [Catholic hospital].” So they very kindly came a day earlier. I think it was on the day that Mum was actually transferred to [Catholic hospital]… So we did that, but that was stressful in itself. (Family caregiver of patient with neurological condition)

It wasn’t an option in [rural town]. So it was only ever available as self-administered. If Dad had to have practitioner assisted, he had to be transported to [major city]. (Family caregiver of patient with cancer)

## Discussion

### Main findings

Most patient experiences of seeking AD were reported by participants to involve institutional objection (17/28 patient cases). These objections were primarily rooted in Catholic religion and/or moral opposition based on a palliative care philosophy. Participants identified three key processes affected: eligibility assessments, medication access, and taking/administration of the medication. Institutional objection occurred across health settings resulting in delays, transfers, choices between progressing an AD application and receiving palliative or other care, and adverse emotional and relationship experiences.

**Fig. 1:**
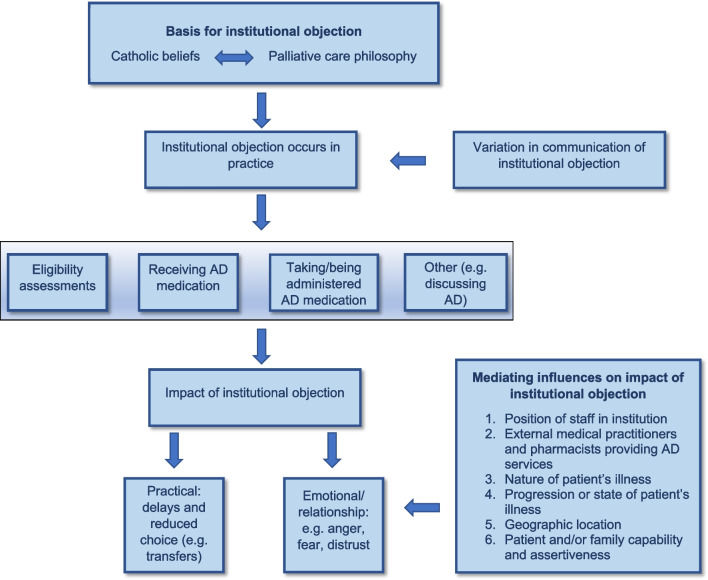
Thematic schema of participants’ perspectives on institutional objection in Victorian assisted dying system

Six mediating influences on institutional objections were identified. Some compound the effect on patients, such as having a particular illness primarily cared for in an objecting institution. Others soften the impact, such as supportive staff in the institution. The schematic relationship between these themes is shown below (Fig. 1).

### Implications of institutional objection as a barrier

The barriers to accessing AD caused by institutional objection can compromise the quality of a patient’s end-of-life experience [9, 32]. Key factors in a “good death” include choice and control in the dying process, and receiving integrated end-of-life care including pain-free status, dignity, and emotional well-being [33]. Yet these findings suggest institutional objection can diminish patient options, require choice between progressing AD and receiving palliative care, and cause emotional discord and stress in a patient’s final days.

Further, the impact of institutional objection on patient access to AD can compound existing access challenges. For example, time delays in the AD approval process can be problematic with patients “racing” to access AD before dying [34]. This is especially so in systems where eligibility criteria require a time period until death, as in Victoria (6 months or 12 months for neurodegenerative conditions) [16]. Delays from institutional objection, even if relatively brief, can threaten access altogether.

Institutional objection also exacerbated challenges for patients with neurological conditions. There were already fewer trained AD doctors in this specialty in Victoria at the time of this research [35], but patient access was reported as being further impeded due to a statewide neurological facility being an objecting institution. Given patients with neurological conditions are a recognised cohort who seek AD [35, 36], this institutional objection is problematic for access.

A linked observation, echoing Wiebe et al.’s [13] findings from Canada, is that these findings reveal a “lottery” of sorts, with some patients better able to navigate institutional objections if the right constellation of mediating factors is present. This creates inequities in care based on inappropriate considerations such as geographical location, illness, practitioners encountered, and available family and other supports.

### Contested permissibility and scope of institutional objection

These findings inform debates about permissibility of institutional objections and, if allowed, their justifiable boundaries. Given the dearth of empirical research on institutional objection, the adverse impacts on patients (and caregivers) in Victoria’s AD system found in this study support at least some limits to institutional power. Further, findings of variability of staff views about AD within objecting institutions may undermine arguments to permit such objections. If safeguarding institutional “conscience” is based on protecting a broad staff consensus, such arguments are undermined by this finding, reflecting studies in other settings [37, 38].

These findings also raise questions about how best to respond to identified harms to patients from institutional objection. Should this be regulated by the state or left to practice, and if formally regulated, what model should be chosen (e.g. conscience absolutism, non-toleration or some form of reasonable accommodation) [3, 10]? There are also questions about how such regulation (if that path is chosen) should be implemented, e.g. through law, policy and/or funding mechanisms. Any regulatory response would also need to consider questions such as whether the intended duration of the care being provided by an institution impacts on its duties, and whether all stages of the AD process should be treated the same. To illustrate, legislation in the Australian state of Queensland imposes higher duties on long-term care institutions such as residential aged care facilities (which are regarded as a person’s home) than on short-term places of care such as hospitals, and treats access to information about AD differently from taking or administering the medication [39].

Our observations are that the current Victorian approach, based on state-issued (optional) policy guidance, is not effective in achieving the objectives of respecting institutional positions while promoting patient access. This “soft regulation” approach appears to have allowed existing power, resource, and information asymmetry to prioritise institutions’ positions over patient choice. Such an outcome is inconsistent with the wider policy goals of the *Voluntary Assisted Dying Act 2017*.[16]

A further concern was significant variation in how institutional objections were expressed. Some institutions objected explicitly, but other participants learned of objections obliquely, through progressive interactions with staff, and sometimes only after a period of time in care. Uncertainty about institutional positions reflects existing literature [40] and is problematic because it makes informed choices about care more difficult for patients and caregivers. Regardless of views about the permissibility of institutional objections to AD, transparency and clear communication of positions are desirable.

### Limitations of the study

This is one of very few empirical studies internationally to examine institutional objection to AD. Importantly, it provides evidence about the adverse impact that institutional objection can have on patients (albeit as reported by family caregivers). However, a limitation of this research is that the perception of caregivers may differ from those of patients as caregivers may be affected by grief, bereavement, and their relationship with the patient they were supporting [25, 26]. However, proxies have been found to be a reliable source of information regarding quality of end-of-life services, demonstrating high concordance with patient views [41].

Another limitation is that the perceptions of our participants reported in this study are based on their experience of interactions with particular health professionals and institutions. Other perspectives are needed and further research with a broad range of key stakeholders is warranted, including to examine wider system issues such as the role played by institutional policies and protocols in managing objections.

Our sample may also be more favourably disposed towards AD, given our recruitment methods which included via patient interest groups. Further, only three patients in this study missed out on AD. More research with this cohort is needed, including whether objections by institutions contribute to a lack of access and the issues of equity to which that gives rise. Additionally, given many patients were still able to access AD *despite* objections by institutions, further investigation is needed as to the reasons for this, including the mediating factors identified in this research (e.g. a capable and assertive patient and/or family caregiver).

Finally, further research is also needed on the intersection between individual conscientious objection and institutional objection, including how one may shape the other. Findings here were that staff positions mediated institutional objections, but more research is needed.

## Conclusion

Institutional objection is a much-debated aspect of AD practice yet is empirically understudied. This research found that in Victoria, it was regularly reported by participants and adversely affected patient and caregiver experience when accessing AD. This occurs in an already procedurally challenging system, particularly given the limited window patients have to apply. Better regulation may be needed to address this issue as the existing policy approach appears to preference institutional positions over patient’s choice given existing power dynamics.

### Supplementary Information

**Additional file 1**. Interview guide.

## Data Availability

The interview guide is available in Additional File 1. The data generated and analysed in this study are not publicly available due to confidentiality undertakings given to research participants as required by the study’s ethics approval. Requests to discuss this should be directed to the corresponding author.

## Abbreviation

AD: Assisted dying

## References

[1] White BP, Willmott L (2021). International perspectives on end-of-life law reform: politics.

[2] Clarke S (2017). Conscientious objection in healthcare: new directions. J Med Ethics.

[3] Wicclair MR (2012). Conscientious Objection in health care: an ethical analysis.

[4] Gilbert D (2020). Faith and/in medicine: religious and conscientious objections to MAiD. Dalhous Law J.

[5] Wolfe ID, Pope TM. Hospital mergers and conscience-based objections—growing threats to access and quality of care. N Engl J Med. 2020;2. 10.1056/NEJMp1917047 32268024

[6] Durland SL (2011). The case against institutional conscience. Notre Dame Law Rev.

[7] Annas GJ (1987). At law: transferring the ethical hot potato. Hastings Cent Rep.

[8] Shadd P, Shadd J (2019). Institutional non-participation in assisted dying: changing the conversation. Bioethics.

[9] Sumner LW (2019). Institutional refusal to offer assisted dying: a response to Shadd and Shadd. Bioethics.

[10] White BP, Willmott L, Close E, Downie J (2021). Legislative options to address institutional objections to voluntary assisted dying in Australia. Univ New South Wales Law J Forum.

[11] Waran E, William L (2020). Navigating the complexities of voluntary assisted dying in palliative care. Med J Aust.

[12] Shaw J, Wiebe E, Nuhn A, Holmes S, Kelly M, Just A (2018). Providing medical assistance in dying: practice perspectives. Can Fam Physician.

[13] Wiebe E, Sum B, Kelly M, Hennawy M (2022). Forced and chosen transfers for medical assistance in dying (MAiD) before and during the COVID 19 pandemic: a mixed methods study. Death Stud.

[14] Holmes S, Wiebe E, Shaw J, Nuhn A, Just A, Kelly M (2018). Exploring the experience of supporting a loved one through a medically assisted death in Canada. Can Fam Physician.

[15] Hashemi N, Amos E, Lokuge B (2021). Quality of bereavement for caregivers of patients who died by medical assistance in dying at home and the factors impacting their experience: a qualitative study. J Palliat Med.

[16] White BP, Del Villar K, Close E, Willmott L (2020). Does the Voluntary Assisted Dying Act 2017 (Vic) reflect its stated policy goals?. Univ New South Wales Law J.

[17] Department of Health. Victoria A. Health services information [Internet]. State Government of Victoria, Australia. 2019. http://www.health.vic.gov.au/patient-care/health-services-information. Accessed 1 Sep 2022.

[18] Close E, Willmott L, Keogh L, White BP. Institutional objection to voluntary assisted dying in Victoria, Australia: an analysis of publicly available policies. J Bioethical Inq. (accepted, forthcoming). 10.1007/s11673-023-10271-6 PMC10624699 37428353

[19] Optimal Regulation of Voluntary Assisted Dying. https://research.qut.edu.au/voluntary-assisted-dying-regulation/. Accessed 25 Jan 2023.

[20] White BP, Willmott L, Close E. Better regulation of end-of-life care: a call for a holistic approach. J Bioeth Inq. 2022;19(4):683–693. 10.1007/s11673-022-10213-8 PMC9908626 36251135

[21] Maxwell JA (2012). A realist approach for qualitative research.

[22] Braun V, Clarke V (2022). thematic analysis: a practical guide.

[23] Byrne D (2022). A worked example of Braun and Clarke’s approach to reflexive thematic analysis. Qual Quant.

[24] Tong A, Sainsbury P, Craig J (2007). Consolidated criteria for reporting qualitative research (COREQ): a 32-item checklist for interviews and focus groups. Int J Qual Health Care.

[25] Addington-Hall J, McPherson C (2001). After-death interviews with surrogates/bereaved family members: some issues of validity. J Pain Symptom Manage.

[26] Williams BR, Woodby LL, Bailey FA, Burgio KL (2008). Identifying and responding to ethical and methodological issues in after-death interviews with next-of-kin. Death Stud.

[27] Willmott L, White BP, Sellars M, Yates P (2021). Participating doctors’ perspectives on the regulation of voluntary assisted dying in Victoria: a qualitative study. Med J Aust.

[28] White BP, Willmott L, Sellars M, Yates P. Prospective oversight and approval of assisted dying cases in Victoria, Australia: a qualitative study of doctors’ perspectives. BMJ Support Palliat Care. 2021. 10.1136/bmjspcare-2021-002972 34092550

[29] Sellars M, White BP, Yates P, Willmott L (2022). Medical practitioners’ views and experiences of being involved in assisted dying in Victoria, Australia: a qualitative interview study among participating doctors. Soc Sci Med.

[30] Braun V, Clarke V (2021). To saturate or not to saturate? Questioning data saturation as a useful concept for thematic analysis and sample-size rationales. Qual Res Sport Exerc Health.

[31] Kitto SC, Chesters J, Grbich C (2008). Quality in qualitative research. Med J Aust.

[32] Sumner LW (2021). Conscientious refusal to provide medically assisted dying. Univ Tor Law J.

[33] Meier EA, Gallegos JV, Thomas LPM, Depp CA, Irwin SA, Jeste DV (2016). Defining a good death (successful dying): literature review and a call for research and public dialogue. Am J Geriatr Psychiatry.

[34] Thangarasa T, Hales S, Tong E, An E, Selby D, Isenberg-Grzeda E (2022). A race to the end: family caregivers’ experience of medical assistance in dying (MAiD)—a qualitative study. J Gen Intern Med.

[35] Voluntary Assisted Dying Review Board. Report of operations: January to June 2021 [Internet]. Safer Care Victoria. 2021. https://www.safercare.vic.gov.au/sites/default/files/2021-08/VADRB%20August%202021%20report%20FINAL.pdf. Accessed 30 Aug 2022.

[36] Emanuel EJ, Onwuteaka-Philipsen BD, Urwin JW, Cohen J (2016). Attitudes and practices of euthanasia and physician-assisted suicide in the United States, Canada, and Europe. JAMA.

[37] Stulberg DB, Lawrence RE, Shattuck J, Curlin FA (2010). Religious hospitals and primary care physicians: conflicts over policies for patient care. J Gen Intern Med.

[38] Freedman LR, Stulberg DB (2013). Conflicts in care for obstetric complications in Catholic hospitals. AJOB Prim Res.

[39] Voluntary Assisted Dying Act 2021 (Qld), Part 6, Division 2.

[40] Takahashi J, Cher A, Sheeder J, Teal S, Guiahi M (2019). Disclosure of religious identity and health care practices on Catholic hospital websites. JAMA.

[41] McPherson CJ, Addington-Hall JM (2003). Judging the quality of care at the end of life: Can proxies provide reliable information?. Soc Sci Med.

