# Supplementary material for: The impact on patients of objections by institutions to assisted dying: a qualitative study of family caregivers’ perceptions
Source: BMC Med Ethics. 2023 Mar 13;24:22. doi: 10.1186/s12910-023-00902-3 (PMC10009962; doi:10.1186/s12910-023-00902-3)
Supplement: Supplementary file 1 — Additional file 1. Interview guide. [file 12910_2023_902_MOESM1_ESM.docx]

**The impact on patients of objections by institutions to assisted dying: a qualitative study of family caregivers’ perceptions**

**Additional File 1**

[This semi-structured interview guide was written for both patients and family caregivers. For generic issues such as the process of seeking voluntary assisted dying, it was written in language for patients but was adapted in the interviews with family caregivers to be expressed as seeking their perception of the patient’s experience of these matters. However, some sections needed to be specifically directed to patients or family caregivers (e.g. after death experiences) and this is indicated by the headings below. Where relevant experiences outside the interview guide were raised, they were also explored.]

**Interview guide**

**Preliminary discussion**

- Introduce interviewers
- Thank you for being able to help this research. Before we get into the detail, can we just first deal with the administrative side of things?
- [Zoom recording, consent and confidentiality discussion]

Thank you very much for agreeing to take part in this interview about voluntary assisted dying. The purpose of the interview is to hear from you, in your own words, your experiences [or those of your family member] of making decisions about voluntary assisted dying. There is a chance you may feel that some of the questions asked are stressful or upsetting. If you do not wish to answer a question, you may skip it and go to the next question, or you may take a break. You may find some questions difficult to answer, however we are interested in whatever information you are able to provide. Please answer the questions in as much or as little detail as you like. Do you have any questions for me before we start the interview?

**About the person seeking VAD (introduction)**

If patient

Before we go on to talk about your experiences of making decisions about voluntary assisted dying, would it be OK if we asked about your illness and where you are now? Are you okay with that?

- Prompts: if appropriate, a chance to understand a bit about their approach to managing their illness and decision-making.

If family

Before we go on to talk about your experiences of making decisions about voluntary assisted dying, would it be possible if you could maybe just give us a short picture of your [family member]? We will talk about this some more – but just a nutshell, even a minute or two, just to help us get to know [family member] first.

**Remaining interview approach**

In terms of our discussion, I know we have a lot to cover and I want to make sure we can capture it all. I have found it often helps if I maybe step through the VAD process in order so you can comment on each part of the process. But I want to finish with some more general and open-ended questions which will also provide a chance for you to tell me other things. Is it OK if we proceed that way and perhaps if am a bit directive to make sure we understand your experience?

[Be prepared though if participant wants to talk about general things first – in which case start with the general questions from the end]

**First discussion of VAD (and first request)**

- When did you first become aware of VAD and that it might be possible? How did this topic come up? (e.g. sources of information) Who did you talk to about this?
- Was it easy to get information you wanted about VAD or were there barriers? Where did you go for information?
- When did you first talk about VAD with a doctor or health professional? How was discussion of this topic received? Were there any barriers or concerns with your doctor or other health professionals?
- Did you know that health professionals can’t raise VAD with you first? What do you think of that law?

**Assessment process [first assessment, second assessment, appointing a contact person, written declaration, permit]**

- What is your recollection of how your eligibility for VAD was assessed?
  - Prompts: Was it straightforward? Challenging? Why? Were any eligibility criteria difficult to satisfy?
  - Who helped and who hindered?
- What parts of the process worked well?
- What parts of the process needed improvement?
- General prompts
  - How did you find the VAD Care Navigator service?
  - Did you ever use telehealth for VAD consultations? Were there issues with travel to avoid telehealth?
  - Did your facility facilitate access to VAD or was it a barrier to access? How?
  - Were you able to find a doctor to coordinate the process, and then a second doctor?
  - Were you able to find witnesses to witness the written declaration?
  - Were you aware that your doctor had to obtain a permit? How did you find the permit process?

**Prescription of medication and provision of VAD for eligible VAD patients [prescription process, self-administration vs practitioner-administration, after death care]**

- Was there a discussion about which method of VAD to use? [self or practitioner administration] Would you have preferred a choice?
- What was your experience of obtaining the medication?
  - Prompts: Was it straightforward? Challenging? Why?
- What aspects of the process for getting the medication work well and what needs improvement?
- How long did the process take from when you started seeking VAD to when you had access to the medication? (explore time from when made formal first request to access to medication as well)
- General prompts
  - What was your experience with the Statewide Pharmacy service?
  - Did your facility facilitate access to VAD or was it a barrier to access? How?

Family members additionally asked these questions:

- What was your experience of accessing and using the medication? [looking for system issues, not clinical ones]
  - Prompts: Was it straightforward? Challenging? Why?
  - Prompts: Preparing the medication, experience of person taking it or doctor administering it
- How did the VAD system work after [family member’s death]?
  - Prompts: Who was the contact person? Why where you/they appointed? How did you/they find undertaking that role? Other issues?
- After the process was all finished, did you have any contact with anyone about how the VAD system worked or initiate yourself providing any feedback (positive or negative)? How it was handled by the institution/health professional?
- Once family member got the medication, how did they feel about that? Why did they go down this path of seeking VAD? (motivation, what they wanted)
- The choice about whether or not to take it, and if did, when to take it. How did they make these decisions and what guided them about the decision to take the medication and its timing?

Patients additionally asked these questions:

- Who is your contact person and why did you appoint them?
- If has medication/or if getting medication: Now that you have the medication (if have), how do you feel about that? Why did you go down this path of seeking VAD? (motivation, what they wanted)
- If has medication/or if getting medication: now/if that you have the medication, the choice about whether or not to take it, and if you do, when to take it – this choice is yours. How will you make these decisions and what will guide you about if and when?
- If you do take the medication, do you have any plans in terms of how that will occur? Who present, how happen, process.

**Sources, operation, navigation and integration of VAD regulation**

- How did you know what process to go through in seeking VAD?
- What did you do, or would you do, if you were unsure of the process or what was permitted or not permitted? Where would you look (or did look) or who would you (or did you) ask? What was your role/your family member’s role in progressing the process – who was “driving” the procedural aspects: the health professionals, patient, family?
  - Prompts: Guided by doctor, VAD Care Navigators, contact point at hospital, advocacy group, other?
  - Prompts: If talk about information sheets or policies etc, how useful?
- Were there any roadblocks? Disagreements? Difficulties accessing VAD? What did you do to get past this?
  - Prompt: Explore especially if one or more doctors refused eligibility for VAD or to participate as will need to navigate system more by themselves.
  - Prompt (if not covered above): Did your facility facilitate access to VAD or was it a barrier to access? How?
  - Prompt (if disagreement or barrier): Explore if they initiated any complaint processes formal or informal and how they did that.
- What would have helped to make navigating the VAD system easier? Prompt: Not just individuals but what is missing in the system?

**Perceptions about VAD regulation generally**

- What was the biggest challenge you faced in navigating the VAD system? How could current system of VAD be improved? What are barriers?
- What was the thing that worked best with the current system of VAD? What other parts of the system worked well? [explore key factors here]
- We have spoken a lot about the *processes* of VAD but what about the rules about who can have access to it in the first place (explain eligibility criteria if needed). Is this the right group who should have access to the law? (especially discuss with patients found not eligible)
- VAD involves a system that has safeguards to ensure only those who are eligible have access to VAD (safeguarding vulnerable and wider community) while facilitating access for those who qualify (choice for terminally ill patients). So a balance between safe processes that ensure only eligible people access VAD – but a system that is workable so people can in fact access VAD. How do you think the current VAD system strikes this balance?
  - From your experience, did you think the processes in the system fulfilled their role to keep ineligible people out? Or did they make it too hard to get through?
- Putting aside the Victorian system, if you could tell those designing a new VAD system who wanted it to be the best system possible, what advice would you have?
  - What principles or values do you think should underpin that system?
  - What features do you think the system should have?
  - This is about guiding people’s behaviour – what is the most effective way to guide people’s in this area?
- Is there anything else you wanted to mention?

**Demographics**

For this research to properly understand how the VAD system is working, we need to make sure we talk to people with diverse experiences and backgrounds. Would it be OK to ask you some questions about yourself and your care (or your family member) so we can understand your perspectives on what we discuss? Apologies if this is a bit mechanical and we will skip over what we already know from our discussion.

Patient

- Age (or age at death)
- Gender
- Where live (city or town)
- Illness type
- Other relevant medical conditions (i.e. comborbidities)
- Cultural aspects can be important to understand in this area [and relevant for eligibility criteria], so also asking participants about ethnic or cultural background, including country of birth. Also ask if any religious identity.
- Primary place where you/family member are/were cared for [make sure includes location VAD received if not in primary place]:
  - Hospital/hospice/RACF/at home/other
  - Public and private sector
  - Religious institution (if so, what religion)
- When sought VAD (rough timing: aiming to know if in first year of system or after that)
- Approved for VAD and used it/ Approved for VAD but didn’t use it/ Not approved for VAD (e.g. died during process or found to be ineligible)
- Relationship/marital status
- Family around patient and involvement in VAD (very brief but aiming to know if support (or opposition) around and who was involved in process)
- Highest level of education
- Occupation

Family member

- Date of birth/age
- Gender
- Relationship with patient [if not already mentioned]

**Wrapping up**

[Discussion about opportunity to review transcript; receiving findings; check-in about well-being and supports/resources for participants]
